# Tumor genomic alterations and their exploratory association with treatment response in recurrent head and neck cancer patients

**DOI:** 10.1038/s41598-026-68381-1

**Published:** 2026-09-09

**Authors:** Olive Beyer, Andrey Loginov, Danielle Arons, Erin Allor, Hannah Thomas, Ishita Gupta, Most Mauluda Akhtar, Rebecca Krc, Ludmila Danilova, Dmitrijs Lvovs, William Silva Mendes, Matthew H. Brown, Kyle Hatten, Matthew Ferris, Jason Molitoris, Phuoc Tran, Ranee Mehra, Daria A. Gaykalova

**Affiliations:** 1 Institute for Genome Sciences, University of Maryland School of Medicine, Baltimore, MD USA; 2 Department of Otorhinolaryngology-Head and Neck Surgery, Marlene & Stewart Greenebaum Comprehensive Cancer Center, University of Maryland Medical Center, Baltimore, MD USA; 3 Icahn School of Medicine at Mount Sinai, New York, NY USA; 4 Department of Hematology/Oncology, Lankenau Medical Center, Wynnewood, PA USA; 5 Department of Radiation Oncology, University of Maryland School of Medicine, Baltimore, MD USA; 6 Department of Oncology, Sidney Kimmel Comprehensive Cancer Center, Johns Hopkins University, Baltimore, MD USA; 7 Department of Oncology, University of Maryland School of Medicine, Baltimore, MD USA; 8 Present Address: Department of Radiation Oncology, Bon Secours St. Francis Cancer Center, Richmond, VA USA; 9 Present Address: Department of Radiation Oncology, Santa Casa de Misericórdia de Itabuna, Bahia, Brazil

**Keywords:** Mutations, Recurrent HNSCC, Patient outcome, Prognosis, Therapy, Cancer, Computational biology and bioinformatics, Genetics, Oncology

## Abstract

While the genetic landscape of treatment-naïve head and neck squamous cell carcinoma (HNSCC) is well characterized, the molecular features of recurrent disease remain less defined. We investigated the genetic alterations in recurrent HNSCC and their associations with overall survival (OS), progression-free survival (PFS), and treatment response. We performed a retrospective analysis of 86 patients with recurrent HNSCC treated at the University of Maryland Medical Center. Clinical data were extracted from electronic health records, and targeted DNA sequencing of tumor samples was performed by Tempus. Genomic profiles were compared with primary HNSCC profiles from The Cancer Genome Atlas (TCGA). Associations between mutations and clinical outcomes were evaluated using Cox proportional hazards models. *TP53* mutations were most prevalent (55%) and significantly correlated with worse OS (HR: 1.77, *p* = 0.033) and poor treatment response (*p* = 0.0067). *CDKN2A* (29%) and *TERT* (26%) mutations occurred more frequently in recurrent tumors compared to primary TCGA tumors. While *CDKN2A* mutations showed better PFS (HR: 0.57, *p* = 0.045), *TERT* mutations showed association with poorer PFS (HR: 1.66, *p* = 0.045) and reduced response to immune therapy (*p* = 0.0854), although the latter did not reach statistical significance. Notably, although in small number, *FGF4* (9%) mutations were associated with significantly poor survival (HR: 2.16, *p* = 0.049) and poorer outcomes following surgery and/or radiotherapy (*p* = 0.0124). However, contradictory to other mutated genes, *PIK3CA* mutations (14%) correlated with better OS (HR: 0.37, *p* = 0.032). Recurrent HNSCC exhibit distinct genomic alterations associated with survival and therapeutic response. These findings are exploratory and hypothesis-generating. Prospective validation in larger, independent cohorts is necessary before these associations can inform treatment decisions.

**Supplementary Information:**

The online version contains supplementary material available at https://doi.org/10.1038/s41598-026-68381-1.

## Introduction

Head and neck squamous cell carcinoma (HNSCC) is the seventh most common cancer worldwide and remains a major cause of cancer-related morbidity and mortality^1^. Despite advances in surgical management, systemic, and radiotherapeutic interventions, the five-year overall survival rate remains about 50%, and up to half of patients develop disease recurrence, associated with significant morbidity and reduced survival^2^. Several clinical factors influence patient outcomes, including gender, tobacco and alcohol consumption, HPV status, and tumor mutational burden^3,4^. Survival outcomes vary substantially by tumor subsite^5^ and stage at diagnosis^6^, with early-stage localized tumors achieving five-year survival rates near 86%, while distant metastatic disease is associated with survival rates of around 40%^6^. Recurrent or metastatic (R/M) HNSCC correlates with poor prognosis, with a median overall survival ranging from 4.3 to 6.7 months, although outcomes have improved modestly with the advent of immune checkpoint inhibitors such as pembrolizumab, which has extended median overall survival to approximately 12 months^7^. These challenges underscore the need to better understand the biology and therapeutic vulnerabilities of recurrent HNSCC. Genomic alterations play an essential role in HNSCC pathogenesis, and are linked to prognosis and response to therapy^8^. Comprehensive genomic profiling of primary HNSCC has identified recurrent alterations in *TP53*,* CDKN2A*,* PIK3CA*,* FAT1*, and *NOTCH1*, and these mutations have informed biomarker development and targeted therapy exploration^9^. Cetuximab, the anti-EGFR antibody, remains the only FDA-approved targeted agent for HNSCC, although several other EGFR-directed agents and combination therapies, including petosemtamab, amivantamab, and ficerafusp alfa, are currently in clinical trials^10–17^. Understanding the genomic landscape of HNSCC is essential, as specific mutations may serve as biomarkers to guide prognosis or predict treatment sensitivity or resistance.

In contrast to primary tumors, the genomic landscape of recurrent HNSCC is less resolved. Recurrent HPV-positive (HPV+) HNSCC tumors acquire molecular features resembling HPV-negative (HPV-) HNSCC with increased *TP53* mutations, whole genome duplication, and 3p deletion^18^. These tumors also exhibit activation of MYC, SRC, and TGF-beta signaling pathways, which correlate with chemoradiation-resistance and worse prognosis^18^. Likewise, mutations in *TP53*,* CDKN2A*,* PIK3CA*, and *TERT* are associated with resistance to radiotherapy and chemotherapy in recurrent HNSCC^19^, further highlighting the clinical importance of understanding the biology of recurrent disease. Beyond genomic profiling, biomarkers such as programmed death-ligand 1 (PD-L1) expression have significant therapeutic implications, as PD-L1-positive tumors demonstrate greater benefit from anti-PD-1 agents^10–17^.

Given the clinical complexity and biological distinctiveness of R/M HNSCC, comprehensive molecular characterization is urgently needed to refine prognostication, improve therapeutic selection, and identify mechanisms of treatment resistance. In the present study, we analyzed the comprehensive genomic landscape of recurrent HNSCC at a single institution and correlated genetic alterations with clinical outcomes including progression free survival (PFS), overall survival (OS), and response to treatment including surgery, radiation, chemotherapy, and immunotherapy. These findings were compared to data from patients with primary HNSCC using The Cancer Genome Atlas (TCGA), thereby expanding current understanding of recurrent HNSCC biology and identifying potential biomarkers that could guide individualized treatment plans.

## Methods

### Cohort curation and comparative genomic analysis

The cohort included retrospective analysis of 86 patients with confirmed post-treatment recurrent HNSCC and corresponding clinical, treatment, and tumor DNA sequencing datasets. Clinical data were collected from the UMB electronic health records for patients treated between 2007 and 2024. All patients in the UMB cohort had recurrent HNSCC originating from a primary HNSCC site and were categorized into five anatomical subsites [oropharynx, oral cavity, hypopharynx, larynx, nasopharynx]. No randomization or blinding was performed in cohort selection, data collection, or downstream analyses. Ethical approval was obtained from the Institutional Review Board (IRB) at the University of Maryland, Baltimore (UMB) (IRB HP-00101297) and the research was performed in accordance with relevant guidelines. Due to the retrospective nature of this analysis and the use of de-identified data, the requirement for written informed consent was waived by the Institutional Review Board (IRB) at the University of Maryland Baltimore (IRB HP-00101297). Research involving human research participants were performed in accordance with the Declaration of Helsinki.

Tumor sequencing and variant calling for 60 tumor samples from 58 patients were performed by Tempus (RRID: SCR_025832)^20^, while the remaining 28 samples underwent sequencing at UMB (RRID: SCR_005837). Among the Tempus-processed specimens, 56 tumors were sequenced using the xT targeted DNA panel, one sample underwent whole exome sequencing (WES) using the xE panel, and three samples were analyzed via liquid biopsy using the xF panel. For two patients, both tissue-based sequencing (xT) and liquid-based sequencing (xF) were available. Variant calling encompassed either 684 genes or 596 genes, depending on the specific panel, with 591 genes in common between the two panels. Liquid biopsy samples included variant calling for 105 genes, all of which were also found in the xT 684 gene panel, and 104 of which were also found in the xT 596 gene panel. The remaining 28 samples were sequenced using WES at UMB, after which variant calls were curated and mapped to the corresponding genes assessed in the Tempus panels to facilitate uniform downstream analysis.

For external comparison and validation, the genomic data from The Cancer Genome Atlas (TCGA) (RRID: SCR_003193) cohort (*n* = 526) were incorporated^21^. The TCGA dataset includes tumors obtained at initial diagnosis, thus presenting genomic landscape of treatment-naïve primary HNSCC. Since the treatment and response data in the TCGA dataset are incomplete, these data were not included in the clinical outcome analysis.

### Demographic data acquisition and statistical analyses

Clinical and demographic data for UMB patients were extracted from the EPIC electronic medical record system. Data were organized and summarized into tables using R Project for Statistical Computing (v4.2) (RRID: SCR_001905) and the *gtsummary* package (v2.0.3) (RRID: SCR_021319). Statistical comparison using the *gtsummary* package (v2.0.3) (RRID: SCR_021319) were performed using Wilcoxon rank sum test (RRID: SCR_021319) for age (continuous variable); Pearson’s Chi-squared test (RRID: SCR_021319) for categorical variables including sex, race, p16 status, cancer type, anatomic stage, prior cancer status, PDL-1 expression, peri-neural invasion (PNI), extranodal extension (ENE), lymphovascular invasion (LVI), response, and treatment type; and Fisher’s exact test (RRID: SCR_021319) for smoking, alcohol consumption, and drug intake. The threshold for significance of p-values was 0.05, except where otherwise noted. Multiple test adjustment was not considered because of the exploratory nature of the study.

PFS analyses across treatment types, response types, and number of treatment attempts were visualized using Kruskal-Wallis box plots generated using survplots.py from Python’s *pysurvtools* (v0.6.2, requires Python v3.9 or later) (https://github.com/GaykalovaLab/pysurvtools*)*^22^. For the Kruskal-Wallis box plots, Time to Progression was defined as the time from the start of treatment until the date of the next recurrence and can include data from multiple recurrences for each patient. Overall differences among groups were assessed using the Kruskal-Wallis H test and displayed above the box plots. The two-sided Mann-Whitney U test was used to calculate pairwise p-values between groups, which were then corrected within each panel for multiple testing using the Benjamini-Hochberg procedure. Only statistically significant adjusted pairwise p-values are reported (*** = *p* < 0.001, ** = *p* < 0.01, * = *p* < 0.05).

All survival analyses were conducted in RStudio (v2025.05.0) (RRID: SCR_000432) using R (v4.5.1, released 2025-06-13) (RRID: SCR_001905). Cox proportional hazards regression analyses were performed using the *survival* (v3.8.6) (RRID: SCR_021137), *survminer* (v0.5.2) (RRID: SCR_021094), and *forestplot* (v3.2.0) (RRID: SCR_023633) packages. Univariate Cox models were used to evaluate the associations between OS and both clinical variables and individual gene mutations. Multivariate Cox regression analyses were also conducted for each variable, controlled for age, sex, anatomic stage, and CBR response .

Parallel univariate Cox analyses were conducted for PFS using the same set of clinical and genomic variables. Subsequently, multivariate PFS models were performed for each clinical variable and gene, adjusting for age, sex, anatomic stage, and CBR response. All scripts used for the Cox proportional hazards regression analysis have been deposited in GitHub (RRID: SCR_002630) (https://github.com/GaykalovaLab/treatment_genes_survival).

### Exploratory decision tree

An exploratory decision tree was constructed using *adaptree.py* from *pysurvtools* (v0.6.2, requires Python (RRID: SCR_024202) v3.9 or later) (https://github.com/GaykalovaLab/pysurvtools*)*^22^ to visualize the hierarchical structure of clinical and genomic factors associated with treatment response. Variables included in the model were selected based on significance in the Cox analysis and Fisher’s Exact Test, with treatment type and outcome controlled for each patient.

The decision tree hyperparameters were optimized through Bayesian optimization, using maximization of average leaf purity as the objective function. This approach allowed the algorithm to iteratively evaluate various tree configurations and identify parameter settings that best separated patients into final subgroups enriched for either CBR Responders (complete response, partial response, or stable disease > 6 months) or CBR Non-Responders (stable disease < 6 months and progression). To limit model complexity and reduce the risk of overfitting, a minimum leaf size of six observations was imposed.

### Data visualization

Data visualization was performed using Python-(RRID: SCR_024202) and R-based tools to illustrate cohort characteristics, mutation patterns, and associations with clinical outcomes.

The Python library *pyswimplot* (v0.6.4) (https://github.com/GaykalovaLab/pyswimplot*)* was used to generate swimmer plots for the UMB cohort, in which patients were color-coded by survival status and annotated by treatment modalities and therapeutic response.

Oncoplots depicting the top 30 most frequently mutated genes in both the UMB and TCGA cohorts were generated using oncoplot.py from the Python library *pysurvtools*. The plots incorporated relevant clinical variables, including sex, smoking, p16 status, cancer type, previous cancer, and initial treatment response^23^.

Variables and gene mutations with p-values ≤ 0.1 in the multivariate Cox proportional hazards models were selected for visualization using the *forestplot* package (v3.2.0) (RRID: SCR_023633). Additionally, Fisher’s exact test plots evaluating associations between mutated genes and treatment types were generated using *survplots.py* from *pysurvtools* (v0.6.2). The threshold for p-value significance in the Fisher’s exact tests was 0.1, and multiple test adjustment was not considered for these plots due to the exploratory nature of the study.

## Results

### Study cohort

To study the genetic mutational landscape of recurrent HNSCC tumors and patient outcomes, we assembled a cohort of 86 patients with histologically confirmed post-treatment recurrent HNSCC. A total of 88 samples were analyzed as two patients contributed two distinct samples collected at different time-points. All patients in this cohort experienced one or more recurrence or progression events with the majority undergoing at least one therapeutic intervention corresponding to each occurrence.

Within the UMB cohort, oral cavity (42%) and oropharyngeal (35%) tumors represented the most common primary sites. Thirty-two patients (37%) exhibited p16 positivity. The median age at the time of sample collection for the study was 63 years (interquartile range 53–70), and the cohort was predominantly male (*n* = 61, 71%) and White (*n* = 59, 69%). A substantial proportion of patients had a history of tobacco exposure (*n* = 49, 57%), while alcohol and drug use were documented in 38% and 13% of patients, respectively (Table 1).

**Table 1:** Clinicopathologic characteristics of patients in UMB and TCGA cohorts.

Characteristic	*n*	UMB, *n* = 86	TCGA, *n* = 526	*p*-value
Age	612	63 (53, 70)	61 (53, 69)	0.9
Sex	612			0.7
Female		25 (29%)	142 (27%)	
Male		61 (71%)	384 (73%)	
Race	612			***< 0.001***
Black		15 (17%)	47 (8.9%)	
Other		12 (14%)	13 (2.5%)	
Unknown		0 (0%)	14 (2.7%)	
White		59 (69%)	452 (86%)	
Smoking	612	49 (57%)	176 (33%)	***< 0.001***
p16	612			***< 0.001***
Negative		54 (63%)	485 (92%)	
Positive		32 (37%)	41 (7.8%)	
Alcohol	601	33 (38%)	351 (68%)	***< 0.001***
Unknown		0	11	
Drugs	86	11 (13%)	NA	
Cancer Type	612			***< 0.001***
Hypopharynx		2 (2.3%)	10 (1.9%)	
Larynx		9 (10%)	117 (22%)	
Nasopharynx		9 (10%)	0 (0%)	
Oral Cavity		36 (42%)	318 (60%)	
Oropharynx		30 (35%)	81 (15%)	
Anatomic Stage	537			***< 0.001***
I		16 (19%)	27 (6.0%)	
II		15 (17%)	73 (16%)	
III		15 (17%)	82 (18%)	
IV		40 (47%)	269 (60%)	
Unknown		0	75	
Prior Cancer	86			
N		65 (76%)	NA	
Y		21 (24%)	NA	
PDL-1 Expression	86			
Unknown		31 (36%)	NA	
Negative		7 (8.1%)	NA	
Positive		48 (56%)	NA	
Lymphovascular Invasion	612			0.5
Unknown		24 (28%)	172 (33%)	
N		37 (43%)	230 (44%)	
Y		25 (29%)	124 (24%)	
Perineural Invasion	612			0.7
Unknown		22 (26%)	158 (30%)	
N		34 (40%)	197 (37%)	
Y		30 (35%)	171 (33%)	
Extranodal Extension	86			
Unknown		29 (34%)	NA	
N		37 (43%)	NA	
Y		20 (23%)	NA	
Initial Treatment Response	86			
Complete Response		51 (59%)	NA	
Partial Response		13 (15%)	NA	
Stable Disease		2 (2.3%)	NA	
Progression		20 (23%)	NA	
Clinical Benefit Rate (CBR)	86			
Responder		66 (77%)	NA	
Non-responder		20 (23%)	NA	
Treatment Type	86			
Chemotherapy		45 (52%)	NA	
Immunotherapy		4 (4.7%)	NA	
Other		37 (43%)	NA	

NA: Not Available.

Significant values are in bold-italics.

Comparison of the UMB recurrent cohort with the TCGA primary HNSCC cohort revealed several significant differences in demographic and clinical characteristics (Table 1). Recurrent HNSCC UMB patients had a significantly higher prevalence of smoking (57% vs. 33%, *p* < 0.001) and a significantly higher number of p16-positive patients (37% vs. 8%, *p* < 0.001). Racial composition also differed significantly between cohorts (*p* < 0.001), with the UMB cohort containing a lower proportion of White patients (69% vs. 86%) and a higher proportion of Black patients (17% vs. 8.9%). Distribution of primary tumor sites also differed; in the UMB cohort, there was a predominance of oral cavity (42%) and oropharyngeal (35%) cancers, while the TCGA cohort was composed primarily of oral cavity (60%), and laryngeal (22%) tumors. In contrast, high-risk pathological features such as lymphovascular invasion and perineural invasion were similar between the two cohorts, with no statistically significant differences between the cohorts (Table 1). Overall, the UMB recurrent HNSCC cohort differs markedly from the TCGA primary HNSCC cohort in demographic and clinical characteristics, while high-risk pathological features remain comparable between the two groups.

Clinical outcomes within the UMB recurrent cohort reflected the aggressive nature of recurrent HNSCC. The median OS time was 40 months from the time of initial diagnosis, with a five-year OS rate of 41.8%. Median follow-up time was 38 months (range: 6 to 198 months). The median PFS was 11 months from the time of initial treatment to first recurrence, consistent with previously reported survival patterns in R/M HNSCC^24^.

### Mutational landscape in recurrent and primary HNSCC

In this exploratory analysis, our aim was to describe patterns that may be associated with patient outcomes rather than to test predefined hypotheses. We first generated a swimmer plot to visualize treatment history, treatment responses, and OS for the first five years after diagnosis across the UMB cohort (Supplementary Fig. 1). Following initial diagnosis, patients received chemotherapy, immunotherapy, radiotherapy, and/or surgery, with documented responses classified as complete response (CR), partial response (PR), stable disease (SD), or progressive disease (PD). The majority of patients underwent multiple lines of therapy corresponding to subsequent progression or recurrence events. Visualization of treatment timelines demonstrated that patients who remained alive five years after diagnosis appeared to show a higher frequency of CRs to initial treatment, whereas deceased patients showed fewer initial CRs and required a greater number of therapeutic attempts over the disease course (Supplementary Fig. 1). Although these findings are descriptive and hypothesis-generating, they are nonetheless consistent with the possibility that early and effective therapeutic response plays a critical role in improving survival outcomes for patients with recurrent HNSCC, whereas a reduction in responsiveness with each subsequent therapy line is predictive of poorer outcomes.

**Fig. 1:**
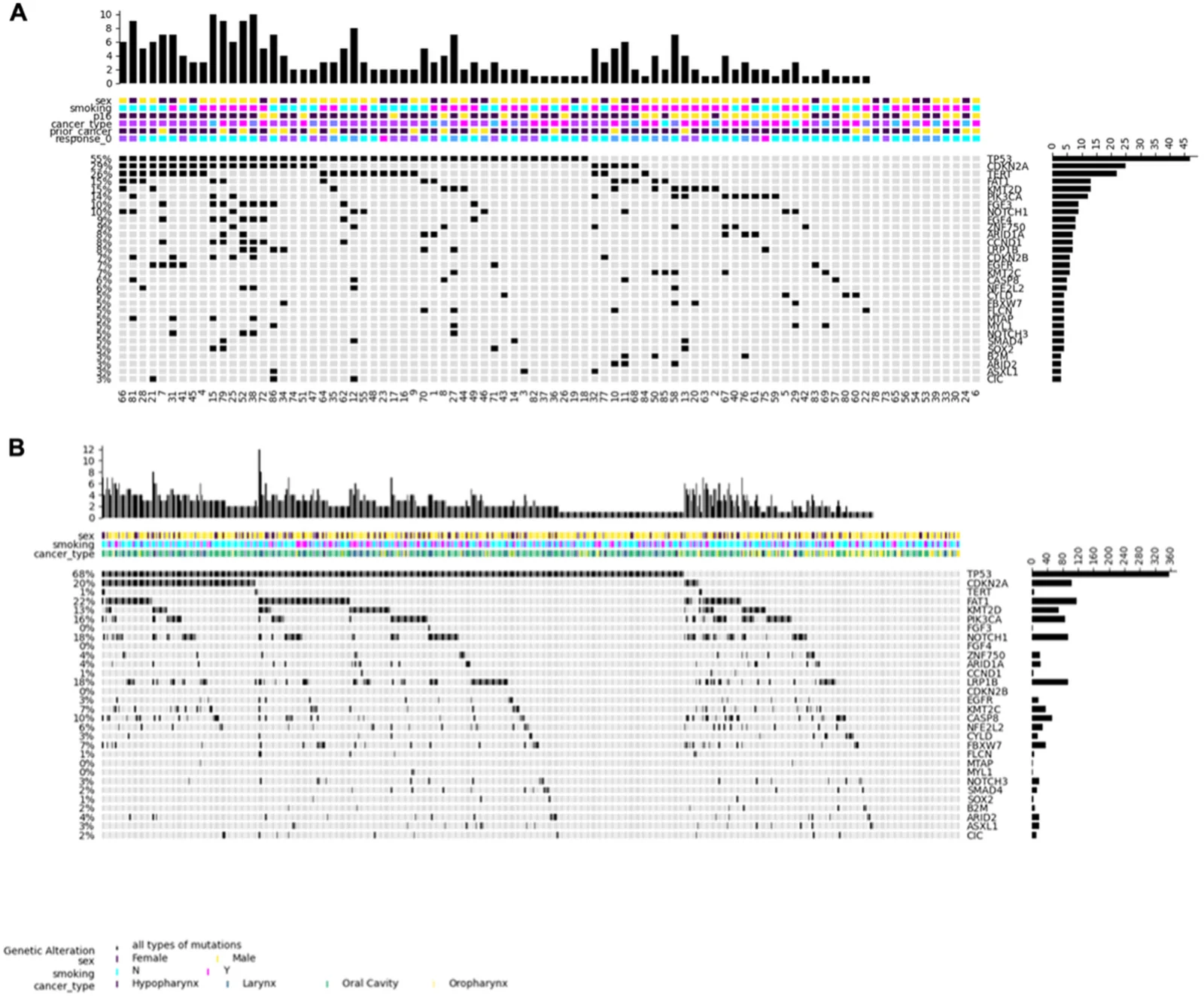
Genetic Landscape for UMB and TCGA cohorts. (**A**). Oncoplot visualization showing the top 30 gene mutations, sex, smoking status, cancer type, p16 status, prior cancer (Y/N), and response to initial therapy for each patient for UMB cohort. Each column represents a patient, and each row represents a different gene. The top barplot indicates the frequency of mutations for each patient. The right barplot notes the frequency of mutations in each gene. (**B**). Oncoplot visualization showing the top 30 gene mutations, sex, smoking status, and cancer type for each patient in the TCGA cohort. Each column represents a patient, and each row represents a different gene. The top barplot indicates the frequency of mutations for each patient. The right barplot notes the frequency of mutations in each gene.

To initialize characterizing the mutational landscape of recurrent HNSCC and compare it with treatment-naïve primary tumors, we generated oncoplots for both the UMB cohort and the TCGA dataset (Fig. 1A and B). In the UMB recurrent cohort, *TP53* was the most frequently mutated gene (55%) (Fig. 1A), consistent with known patterns in HNSCC. However, several notable differences emerged when compared with the TCGA cohort. Recurrent tumors demonstrated highest mutation rates in *CDKN2A* (29%), *TERT* (26%), *FAT1* (15%), *KMT2D* (15%), and *PIK3CA* (14%) (Fig. 1A). Additionally, co-mutation patterns revealed a dense clustering of mutations involving *TP53*,* CDKN2A*,* TERT*, and *KMT2D* (Fig. 1A). Annotation tracks for sex, smoking, prior cancer, response, and tumor site showed that mutations were broadly distributed across clinical subgroups, an observation consistent with the heterogeneous nature of recurrent disease. In contrast, TCGA primary tumors exhibited a distinct pattern. While *TP53* remained the most frequently mutated gene (68%), primary tumors showed the highest mutation frequencies in *FAT1* (22%), *CDKN2A* (20%), *NOTCH1* (18%), *LRP1B* (18%), and *PIK3CA* (16%) (Fig. 1B). Mutation burden was similarly heterogeneous in treatment-naïve HNSCC samples (TCGA). Since the two cohorts were assembled and sequenced independently, these comparisons are intended to be descriptive and to generate hypotheses for formal, statistically powered testing rather than to establish definitive differences between recurrent and primary disease.

### Association between gene mutations and treatment response

**Fig. 2:**
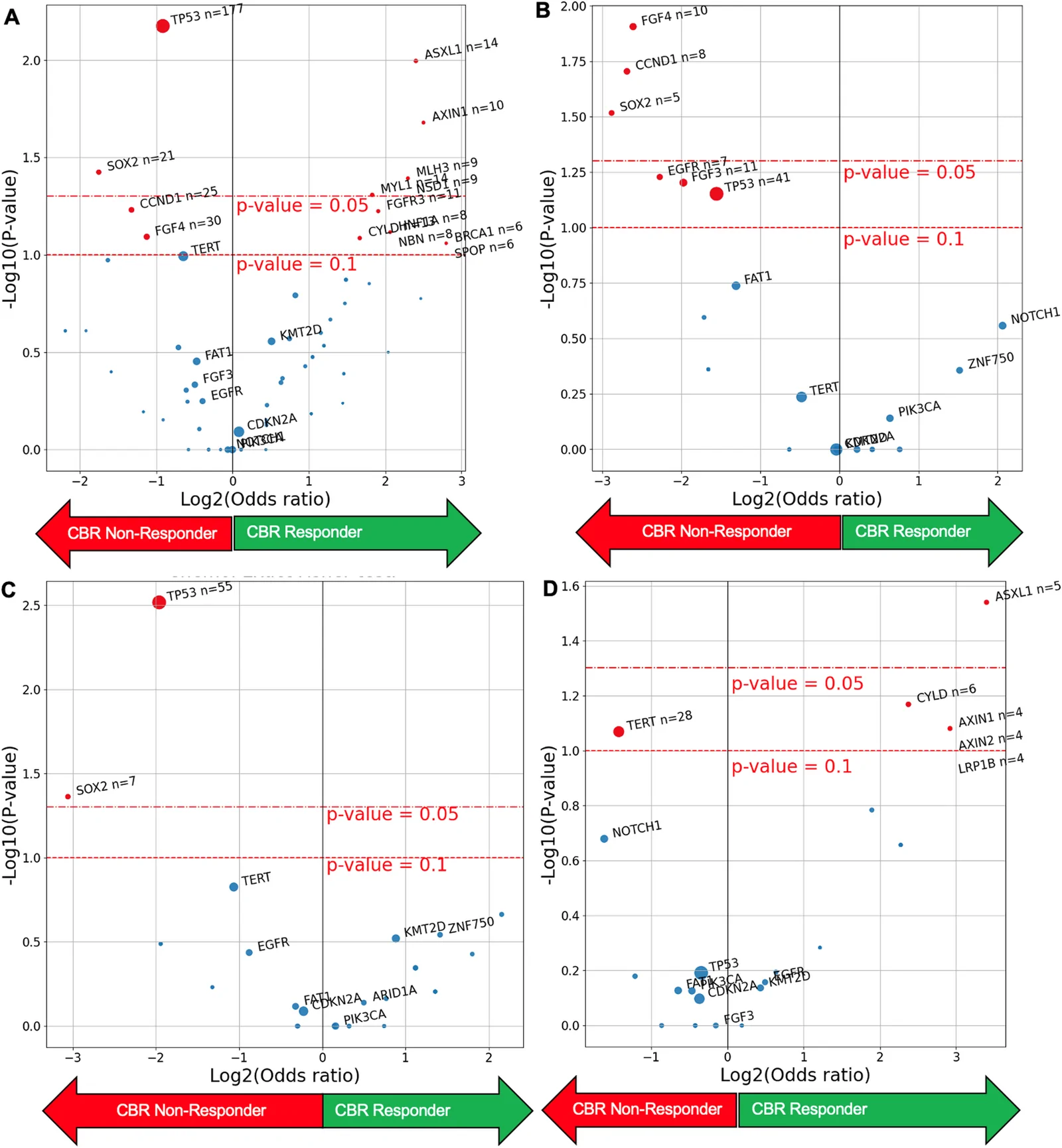
Fisher’s exact test of the relation between mutations and treatment response. Each mutated gene is represented by a point on the scatter plot and the most frequently mutated genes are labeled to indicate the gene they represent. The X-axis represents the logarithm of the odds ratio for disease response, with log_2_(OR) < 0 indicating CBR non-responders with stable disease < 6 months or progressed disease. (**A**). Association between genetic mutations and disease response to all types of treatments. (**B**). Association between genetic mutations and disease response to surgery and/or radiation treatment. (**C**). Association between genetic mutations and disease response to chemotherapy. (**D**). Association between genetic mutations and disease response to immunotherapy.

To investigate how tumor genomics might relate to therapeutic outcomes in recurrent HNSCC, we assessed the association between individual gene mutations and CBR using Fisher’s Exact tests across all treatments combined and within specific therapeutic categories (Fig. 2). Analyses were conducted across all treatment types together (Fig. 2A) and within individual treatment categories, including surgery and/or radiotherapy (Fig. 2B), chemotherapy (Fig. 2C), and immunotherapy (Fig. 2D). However, these analyses were exploratory and were not adjusted for multiple comparisons, so the p-values reported below should be read as nominal and hypothesis-generating rather than as confirmatory evidence. Multiple recurrence and treatment observations may be included for each patient, however, right-censored observations from patients who were alive at the time of data collection are excluded from this analysis.

When all treatment modalities were considered together, several alterations appeared to associate with clinical response (Fig. 2A). Genetic mutations in *TP53* (*p* = 0.0067) and *SOX2* (*p* = 0.0376) were significantly enriched among non-responders, with *CCND1* (*p* = 0.0588) and *FGF4* (*p* = 0.0807) showing a similar trend that did not reach a conventional significance (Fig. 2A) (Supplementary Table 1). In contrast, mutations in *ASXL1* (*p* = 0.0101), *AXIN1* (*p* = 0.0209), *MLH3* (*p* = 0.0405), *NSD1* (*p* = 0.0405), and *MYL1* (*p* = 0.0492) correlated with better CBR response, as well as *FGFR3* (*p* = 0.0597), *HNF1A* (*p* = 0.0765), *NBN* (*p* = 0.0765), *CYLD* (*p* = 0.082), *BRCA1* (*p* = 0.0872), and *SPOP* (*p* = 0.0872) (Fig. 2A) (Supplementary Table 1).

Within the subset of patients treated with surgery and/or radiation, no genetic mutations appeared to associate with enhanced therapeutic benefit (Fig. 2B). However, mutations in *FGF4* (*p* = 0.0124), *CCND1* (*p* = 0.0197), and *SOX2* (*p* = 0.0304), were associated with a worse response to surgery/radiotherapy, and *EGFR* (*p* = 0.059), *FGF3* (*p* = 0.0626), and *TP53* (*p* = 0.0703) trended in the same direction (Fig. 2B) (Supplementary Table 1). In contrast, *NOTCH1*,* ZNF750*, and *PIK3CA* appeared to trend towards improved local-therapy response, although significance was not reached (Fig. 2B). Chemotherapy-specific analysis pointed to *TP53* (*p* = 0.003) and *SOX2* (*p* = 0.0433) as the alterations most closely associated with poor therapeutic response (Fig. 2C). Among patients treated with immunotherapy, mutations in *ASXL1* were associated with a better response (*p* = 0.0289), while *CYLD* (*p* = 0.0679), *AXIN1* (*p* = 0.0831), *AXIN2* (*p* = 0.0831), and *LRP1B* (*p* = 0.0831) showed the same trend. Mutations in *TERT* (*p* = 0.0854) were associated with worse response (Fig. 2D) (Supplementary Table 1). We note that these subgroup analyses rest on small numbers of patients per treatment category, limiting statistical power. Consequently, these findings should be interpreted as exploratory and hypothesis-generating, providing preliminary observations that warrant validation in larger, independent cohorts.

**Fig. 3:**
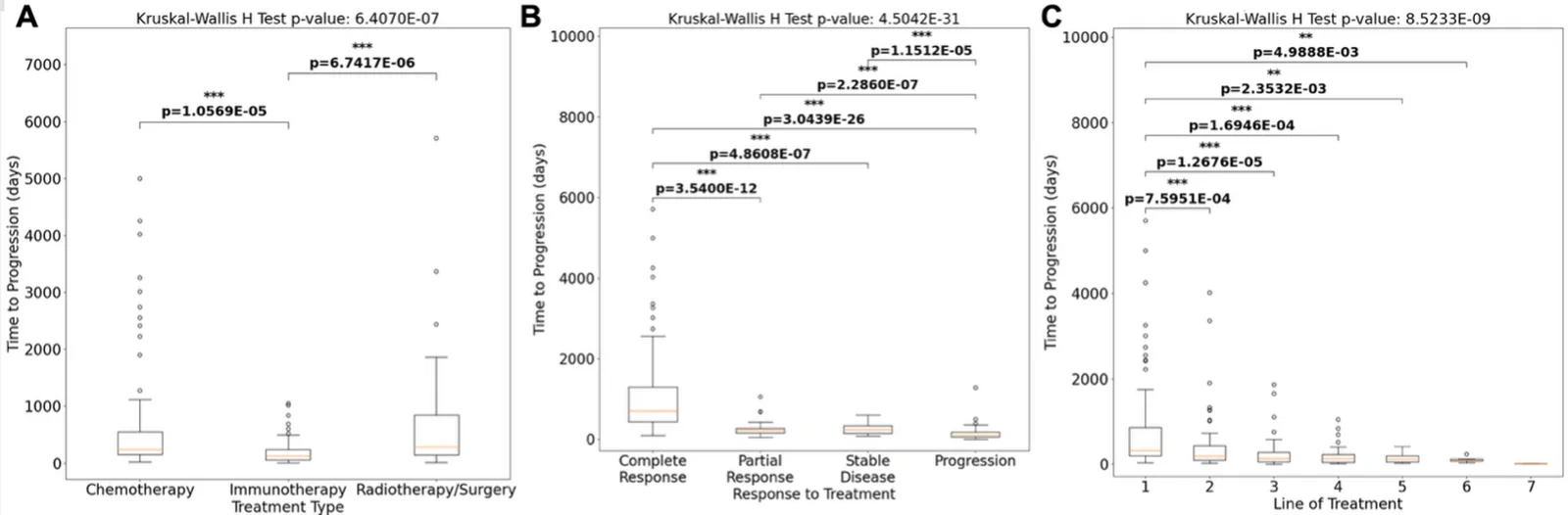
Progression free survival for treatment and response of UMB cohort. Kruskal-Wallis H test indicates differences in progression-free time in days on the Y-axis in relation to (**A**). treatment type, with significant pairwise p-values shown on the plot (Kruskal-Wallis H-test p-value = 6.4070E-7). (**B**) response to treatment, with significant pairwise p-values shown on the plot (Kruskal-Wallis H-test p-value = 4.5042E-31). (**C**). number of prior lines of treatment, with significant pairwise p-values shown on the plot (Kruskal-Wallis H-test p-value = 8.5233E-9).

Together, these findings are consistent with the hypothesis that treatment response in recurrent HNSCC reflects an interplay between therapeutic modality and tumor genomic profile. In this cohort, radiotherapy and surgery were associated with the longest PFS (Fig. 3A), while complete response to treatment showed the strongest association with favorable outcomes (Fig. 3B). Conversely, recurrent tumors harboring genetic mutations in *TP53*, *SOX2*, *CCND1*, and *FGF4* appeared to respond less across multiple therapies. These patterns influence the hypothesis that integrating genomic profiling into therapeutic decision-making could help optimize personalized treatment strategies in recurrent HNSCC, a possibility that would need to be tested in larger, adequately powered, and ideally prospective cohorts.

### Effect of treatment and response on PFS

We next used the Time to Progression for each recurrence to evaluate PFS for three aspects of clinical management: (1) the type of treatment received (categorized as surgery/radiation, chemotherapy, or immunotherapy), (2) the number of treatment attempts, and (3) the response to treatment. Time to Progression was defined as the time from the start of treatment until the next recurrence and can include multiple observations for each patient as they go through multiple treatment and recurrence cycles. Treatment modality was significantly associated with a difference in PFS (*p* = 6.4070E-7) (Fig. 3A). Thus, patients treated with radiotherapy and/or surgery exhibited the longest PFS, while those treated with chemotherapy produced intermediate outcomes. Patients who received immunotherapy treatment demonstrated the shortest PFS (Fig. 3A). Clinical response to the treatment strongly correlates with PFS (*p* = 4.5042E-31) (Fig. 3B). Patients achieving a CR had markedly longer PFS than those with PR, SD, or PD (Fig. 3B). The stepwise decline in PFS from CR to PR, SD, and PD underscores the prognostic importance of treatment efficacy. Additionally, PFS decreased significantly with each subsequent line of therapy (*p* = 8.5233E-9) (Fig. 3C). The first-line treatment attempt exhibited the longest PFS (Fig. 3C), while subsequent attempts yielded progressively shorter intervals. Together, the data demonstrate that PFS in recurrent HNSCC is shaped by the treatment type, response to therapy, and the accumulation of prior treatment attempts.

Treatment distribution varies significantly across lines of therapy, and we have used a bar graph to visualize how often each treatment type was used at specific treatment attempts (Supplementary Fig. 3). Chemotherapy was used more frequently as first-line treatment (Supplementary Fig. 3). Surgery and radiotherapy were also commonly administered in early treatment attempts (Supplementary Fig. 3). In contrast, immunotherapy was rarely used as a first-line therapy compared to chemotherapy and surgery/radiation (Supplementary Fig. 3). However, immunotherapy became more common in later treatment stages (2nd to 4th lines of therapy) (Supplementary Fig. 3).

Treatment distribution across lines of therapy is a limitation in comparing treatment types for all lines of treatment, and we have validated the findings from Fig. 3A that patients who received immunotherapy had the shortest PFS with a similar analysis restricted only to the first line of treatment, showing the same trend (Supplementary Fig. 2A). Due to limited statistical power because immunotherapy was administered in this cohort primarily in the recurrent/metastatic setting, significance is not reached when restricted to first line of treatment. This analysis does not account for clinical differences between patients, such as anatomical stage, which likely also play a role in PFS. In contrast, comparing PFS and the response to first line of treatment only does retain some significance, with CR showing a strong correlation with PFS (*p* = 1.7098E-9) (Supplementary Fig. 2B). Comparing the PFS for chemotherapy administered as a first line or recurrent treatment indicates a significantly improved PFS for initial treatment with chemotherapy, compared to chemotherapy administered in later lines of treatment (*p* = 1.4306E-3) (Supplementary Fig. 2C).

### Exploratory analysis of clinical and genetic correlates of survival outcomes

To explore potential associations between clinical and genetic variables and survival outcomes in recurrent HNSCC, we performed univariate and multivariate Cox proportional hazards analyses for both OS and PFS.

**Fig. 4:**
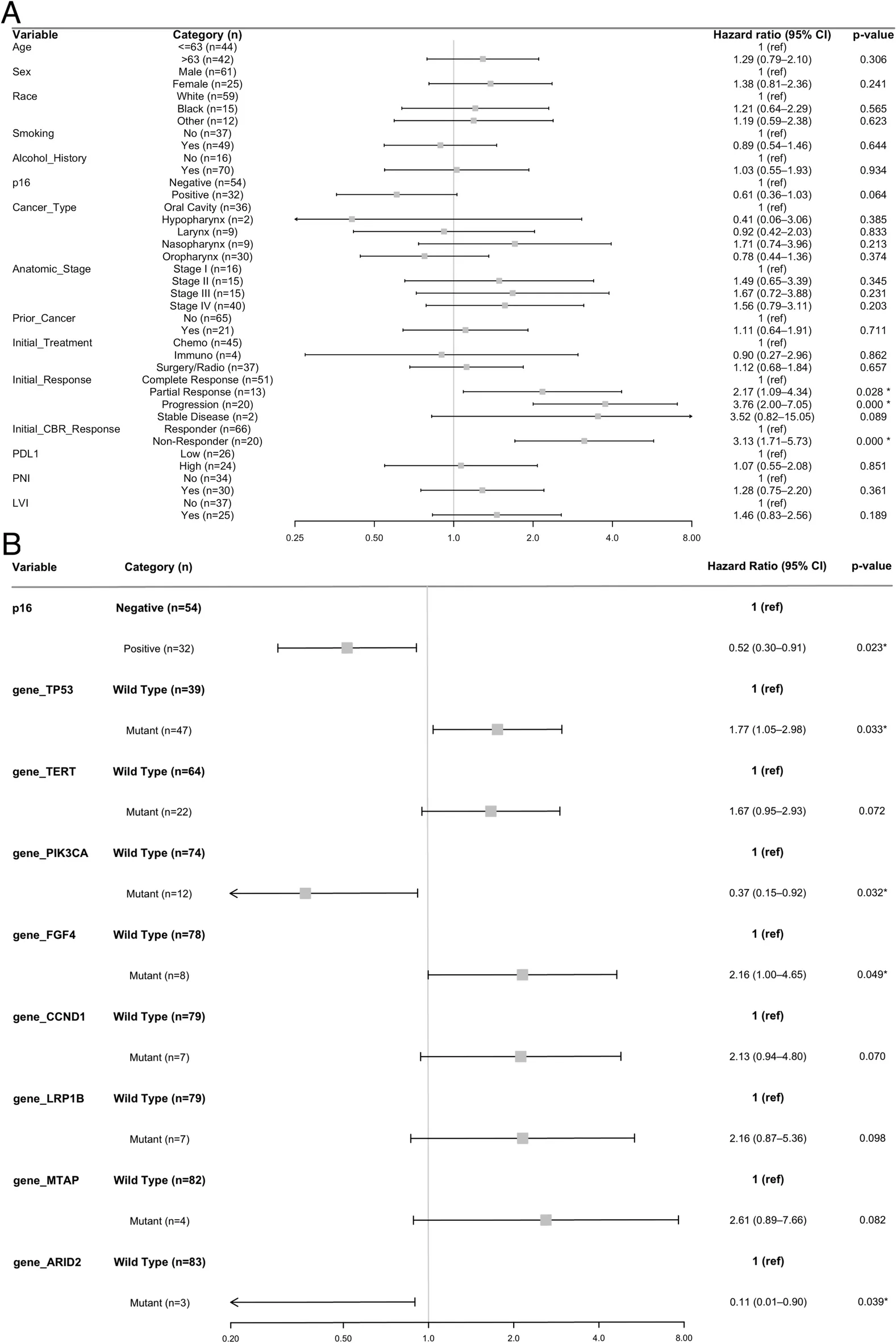

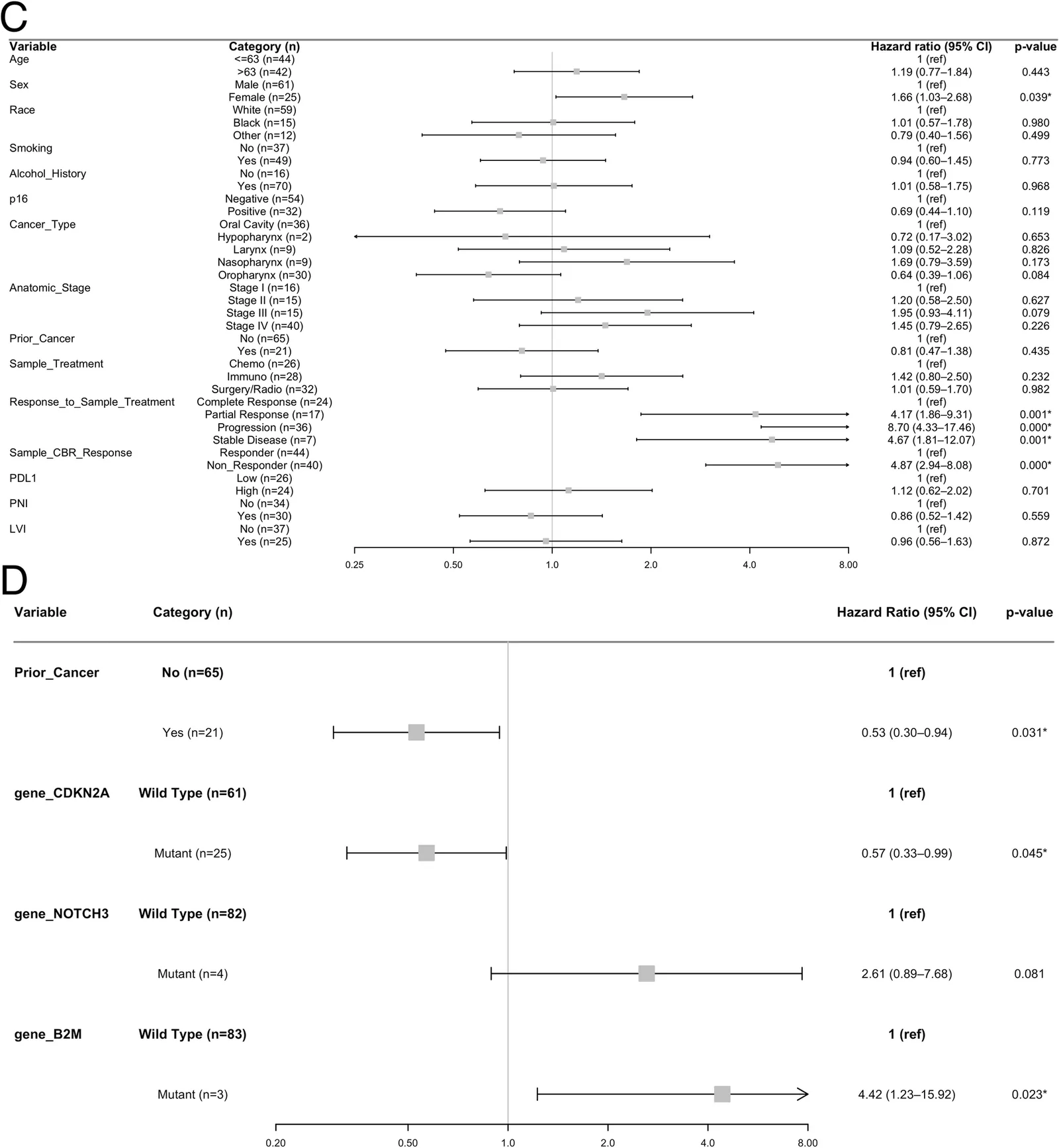
Cox regression analysis of factors associated with (**A** and **B**) overall survival and (**C** and **D**) progression-free survival in recurrent HNSCC. (**A**). Univariate Cox regression analysis of clinical variables. Hazard ratios (HRs) with 95% confidence intervals (CIs) are shown for each clinical factor. (**B**). Multivariate Cox regression analysis of gene mutations. The model was adjusted for age, sex, anatomic stage, and CBR response. (**C**). Univariate Cox regression analysis of clinical factors associated with recurrence. (**D**). Multivariate Cox regression analysis of gene mutations. The model was adjusted for age, sex, anatomic stage, and CBR response.

For evaluation of OS, only one observation is included for each patient. The initial treatment to first recurrence period is considered for the treatment and response variables. Several clinical variables showed associations with OS (Fig. 4A). Patients older than 63 years showed a slightly higher estimated mortality risk compared to those aged 63 years or younger (reference group), although the association did not reach statistical significance (HR: 1.29, 95% CI: 0.79–2.10, *p* = 0.306). Female patients showed the same trend toward poorer survival compared to male patients but also did not reach statistical significance (reference group; HR: 1.38, 95% CI: 0.81–2.36, *p* = 0.241). Patients with p16 positive tumors appeared to have a slightly lower estimated mortality risk compared to patients with p16 negative tumors (reference group), with a borderline p-value that did approach statistical significance (HR: 0.61, 95% CI: 0.36–1.03, *p* = 0.064). Treatment response was among the variables most consistently associated with survival; relative to complete response (reference group), both partial response (HR: 2.17, 95% CI: 1.09–4.34, *p* = 0.028), and progression (HR: 3.76, 95% CI: 2.00–7.05, *p* < 0.001) were associated with higher mortality risk. Similarly, CBR non-responders showed higher estimated mortality than responders (reference group; HR: 3.13, 95% CI: 1.71–5.73, *p* < 0.001). Anatomic stage suggested a non-significant trend towards higher risk with advancing stage, while smoking status and alcohol use were not significantly associated with OS. Among the genes altered in the UMB cohort, mutations in *TP53* (HR: 1.72, 95% CI: 1.04–2.82, *p* = 0.033) were significantly associated with a higher mortality risk, while *ARID2* mutations (HR: 0.12, 95% CI: 0.02–0.90, *p* = 0.039) showed a significantly lower mortality risk for OS compared to the wild type (reference group) (Supplementary Fig. 4A).

In the multivariate model adjusted for age, sex, anatomic stage, and initial treatment CBR response (Fig. 4B), one clinical factor and several genomic alterations were independently associated with OS. Tumors positive for p16 were associated with improved survival compared to p16 negative tumors (reference group; HR: 0.52, 95% CI: 0.30–0.91, *p* = 0.023). Mutations in *PIK3CA* (HR: 0.37, 95% CI: 0.15–0.92, *p* = 0.032) and *ARID2* (HR: 0.11, 95% CI: 0.01–0.90, *p* = 0.039) were significantly associated with improved survival compared to the wild type (reference group), whereas mutations in *TP53* (HR: 1.77, 95% CI: 1.05–2.98, *p* = 0.033) and *FGF4* (HR: 2.16, 95% CI: 1.00-4.65, *p* = 0.049) were associated with worse survival. *TERT* mutation showed a borderline association with better OS (HR: 1.67, 95% CI: 0.95–2.93, *p* = 0.072), along with mutations in *CCND1* (HR: 2.13, 95% CI: 0.94–4.80, *p* = 0.070), *LRP1B* (HR: 2.16, 95% CI: 0.87–5.36, *p* = 0.098), and *MTAP* (HR: 2.61, 95% CI: 0.89–7.66, *p* = 0.082). Together, these findings raise the possibility that specific genetic alterations carry distinct prognostic signals in recurrent HNSCC, a hypothesis that needs further validation in larger, independent cohorts.

Several clinical factors were also associated with PFS (Fig. 4C). For this analysis, only one observation is considered for each patient. We selected the treatment to next recurrence period that is closest in time to the tumor sample collection. Female patients had a higher estimated risk of recurrence than male patients (HR: 1.66, 95% CI: 1.03–2.68, *p* = 0.039). Consistent with the OS findings, patients with partial response (HR: 4.17, 95% CI: 1.86–9.31, *p* = 0.001), disease progression (HR: 8.70, 95% CI: 4.33–17.46, *p* < 0.001), or stable disease (HR: 4.67, 95% CI: 1.81–12.07, *p* = 0.001) following therapy showed a higher risk of recurrence compared to those with a complete response (reference group), and CBR non-responders demonstrated worse PFS compared to responders (reference group; HR: 4.87, 95% CI: 2.94–8.08, *p* < 0.001). For PFS, mutations in *TERT* (HR: 1.66, 95% CI: 1.01–2.72, *p* = 0.045) and *EGFR* (HR: 2.58, 95% CI: 1.10–6.03, *p* = 0.029) reached statistical significance as a predictor of worse PFS relative to the wild type (reference group) in univariate Cox analysis (Supplementary Fig. 4B).

In the multivariate model for PFS, adjusted for age, sex, anatomic stage, and CBR response, a few variables did show statistically significant associations with PFS (Fig. 4D). Patients with prior cancer had a lower estimated risk of recurrence compared to those with no prior cancer history (HR: 0.53, 95% CI: 0.30–0.94, *p* = 0.031). Mutations in *B2M* showed an association with increased recurrence risk (HR: 4.42, 95% CI: 1.23–15.92, *p* = 0.023), while *CDKN2A* mutations showed a statistically significant trend towards improved survival (HR: 0.57, 95% CI: 0.33–0.99). *NOTCH3* mutations showed a trend towards increased estimated recurrence risk compared to wild type that did not reach significance (HR: 2.61, 95% CI: 0.89–7.68, *p* = 0.081). No other variables reached statistical significance in the adjusted model.

Overall, these exploratory analyses suggest that clinical response to treatment may be one of the more consistent correlates of both OS and PFS in recurrent HNSCC, while certain genomic alterations, such as *TERT* and *NOTCH3* mutations, may also contribute to recurrence risk. These observations are hypothesis-generating and warrant further investigation in larger, prospectively designed studies.

### Exploratory decision tree analysis of response patterns

To assess how the combined predictive value of the clinical and genomic features associated with treatment outcome to distinguish CBR response to treatment, we constructed a decision tree model as an exploratory, hypothesis-generating analysis rather than a validated predictive tool. Variables incorporated into the model were selected based on significance in association with survival and response to treatments as seen in the data presented thus far. Variables included both clinical factors (sex, cancer type, treatment group, and anatomic stage) and commonly mutated genes associated with survival and treatment response (*TP53*, *ASXL1*, *AXIN1*, *MYL1*, *SOX2*, *FGF4*, *ARID2*, *CDKN2A*, *PIK3CA*, *MTAP*, *CCND1*, and *TERT)*. To avoid over-representation of repeated outcomes within the same individual, identical treatment-response combinations from a single patient were counted once, however, non-identical treatment-response combinations from the same patient were included. Treatment type itself was excluded as a factor to prevent confounding due to its strong linkage with several included clinical variables.

**Fig. 5:**
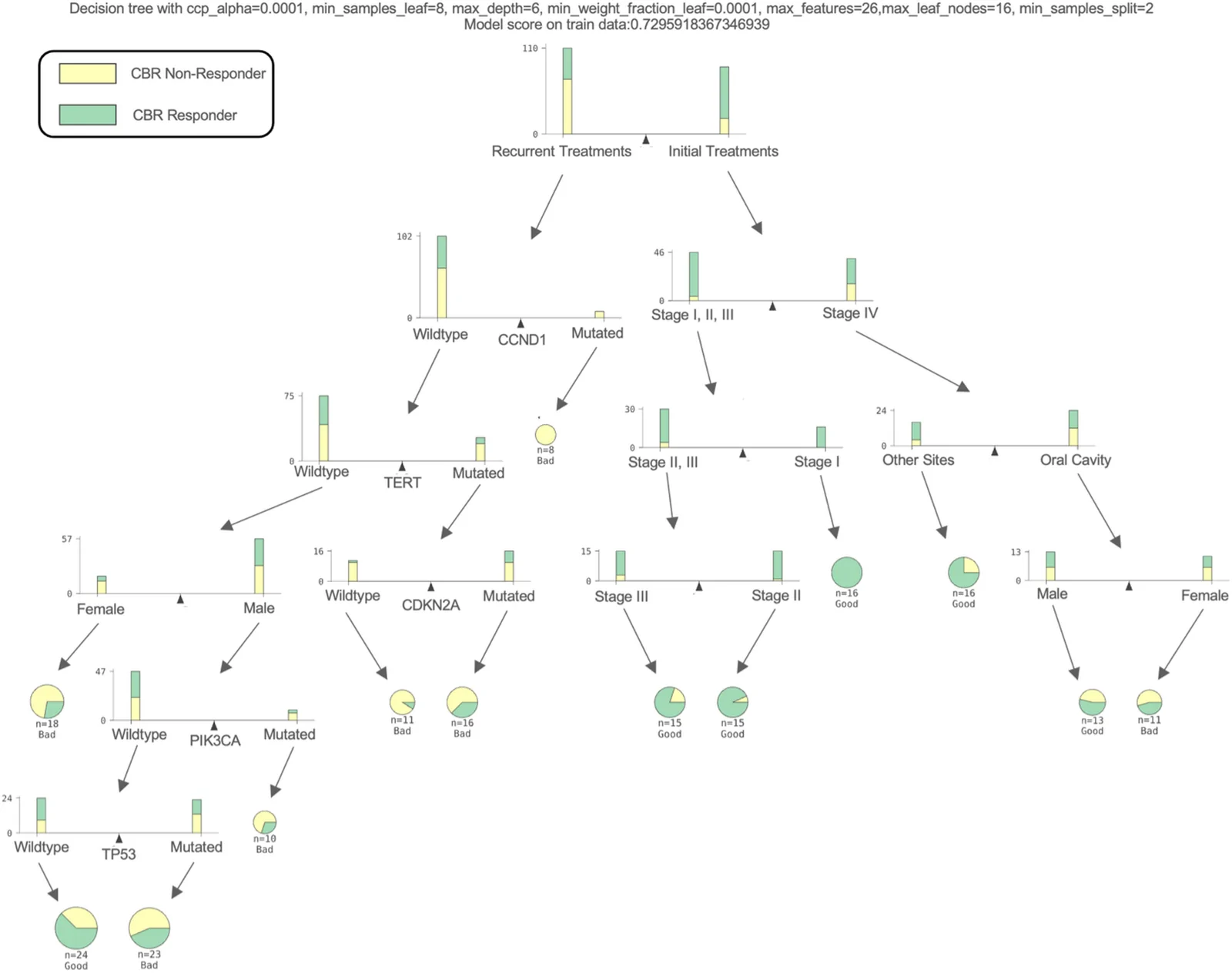
Decision tree predicting clinical benefit response (CBR) based on clinical and genomic variables in recurrent HNSCC. Numbers represent treatment cycles. CBR Responders (shown in green) had complete response, partial response, or stable disease > 6 months and CBR Non-Responders (shown in yellow) had stable disease < 6 months and progression.

The resulting decision tree model accurately predicted CBR in 73% of cases across our cohort (Fig. 5). As no independent test set or cross-validation was applied, this figure represents apparent accuracy on the training data and reflects the fit of the model to the observed cohort rather than its performance on unseen cases. The patterns described below should therefore be read as exploratory observations that require validation in independent cohorts before any predictive claim can be made.

The primary split (top of the tree) separated “Initial Treatments” from “Recurrent Treatments” for recurrent HNSCC, indicating that treatment timing is a major correlate of therapeutic outcome in recurrent HNSCC. Within the initial treatment factor (the first right branch of the tree), anatomic stage emerged as the dominant determinant of response, with patients with early-stage disease generally associated with better responses to therapy. Notably, all patients with anatomic stage I tumors at their initial diagnosis had good outcomes. For stage IV patients, anatomic site was the next dividing factor, with oral cavity as the site generally leading to worse outcomes than all other sites. Sex further stratified outcomes within the initial-treatment group, with female patients less likely to benefit from initial treatment than male patients.

In contrast, features associated with response shifted considerably in the recurrent treatment branch, and there was a stronger association with genetic mutations (Fig. 5). Notably, all mutations in *CCND1* were associated with poor response in the recurrent group, while patients with wild-type *CCND1* and *TERT* mutations showed overall poor response regardless of *CDKN2A* mutation status. Among *CCND1-* and *TERT-*wild-type tumors, additional stratification by sex and *PIK3CA* and *TP53* status further refined response pattern, with female patients generally showing poor outcomes and mutations in both *PIK3CA* and *TP53* associated with poor response, while wild-type *TP53* showed better response. In addition, anatomic stage and tumor site were no longer informative features in this branch.

Overall, the decision tree illustrates how the clinical and genomic features associated with response appear to differ across treatment timelines within this cohort. While initial treatment status is shaped primarily by anatomic stage and tumor site, the recurrent treatment branch was organized around specific genomic alterations (*CCND1*, *TERT*, *CDKN2A*, *PIK3CA*, and *TP53*). These exploratory findings underscore the value of integrating genomic profiling with clinical parameters, particularly for recurrent head and neck cancers, and provide hypotheses to refine personalized treatment strategies in recurrent HNSCC, which will need to be tested and validated in larger, independent cohorts.

## Discussion

In this single-institution study of recurrent HNSCC, we set out to examine how clinical characteristics and tumor genomic alterations relate together to OS, PFS and treatment response. To set these observations within a wider frame, we compared the mutational landscape of our cohort with the treatment-naïve primary HNSCC tumors from TCGA. Collectively, these analyses underscore the distinct biology of recurrent disease and highlight several genetic and clinical factors that can play a role to guide prognostication and therapeutic decision making in recurrent HNSCC.

Among the genetic alterations evaluated, *TP53* mutations correlated with worse OS in the UMB cohort and emerged as one of the stronger correlates of poor OS. This finding is consistent with the well-established role of *TP53* in maintaining genomic integrity; loss of p53 function through mutation disrupts these protective mechanisms, enabling genomic instability, uncontrolled proliferation, and tumor progression^25^. Consistent with these biological effects, a previous meta-analysis showed that *TP53* mutations are linked to worse OS, disease‑specific survival, and PFS^26,27^. In our study, we also found mutations in *LRP1B*, and *MTAP* to associate with poor OS. A recent study in a TCGA-HNSCC cohort showed that 25% of patients had a mutation in *LRP1B*, which correlated with poorer OS^28^. Prior studies also report *LRP1B* alterations to enhance cell proliferation, colony formation, and cell migration, while loss of *LRP1B* increases resistance to chemotherapy and radiotherapy^29^. *MTAP* deficiency, often co-occurring with *CDKN2A*/*CDKN2B* loss, is linked to worse disease-specific survival and distant metastasis-free survival and immunomodulatory effects across several malignancies^33,34^. Our findings suggest that these genes could function as potential prognostic biomarkers in recurrent HNSCC and may contribute to treatment resistance, a hypothesis that needs further validation.

In contrast, mutations in *PIK3CA* were associated with improved OS in our cohort. *PIK3CA* mutations, present in 14% of UMB patients, are canonical oncogenic drivers, yet they are also linked to improved invasive disease-free survival^35,36^. Concordant to our findings, Zhang et al. (2020)^27^ revealed *PIK3CA* mutations, as well as age > 65 years, were independently associated with improved OS in the HNSCC cohort from the Memorial Sloan Kettering Cancer Center (MSKCC) and TCGA cohorts. The study reported the *PIK3CA-* mutant tumors exhibited higher tumor mutational burden, increased expression of immune checkpoint molecules, and a more immune-active tumor microenvironment, microsatellite instability, and frequent DNA damage repair gene alterations; all of which can contribute to improved responsiveness to immune checkpoint inhibitors^37^. Although not conclusive, the presence of *PIK3CA* appearing protective in our cohort can be explained from the perspectives of tumor mutational burden and PD-L1 expression and highlights the need for further investigation of its value as a potential prognostic marker in recurrent HNSCC.

With respect to PFS, *TERT* and *NOTCH3* mutations were associated with worse PFS in the UMB cohort. *TERT* plays a key role in maintaining telomere length and enabling cellular immortality^39^. Lyu et al. (2023)^39^ found *TERT* alterations to be significantly correlated with poor prognosis in head and neck cancers. Furthermore, *NOTCH3*, a component of the Notch signaling pathway involved in cell differentiation and survival, is also implicated in HNSCC progression. Suppression of *NOTCH3* improved survival in mouse models of metastatic head and neck cancer, supporting its potential role in disease aggressiveness^40^. A previous study with a subset of our UMB cohort reported *NOTCH4*, another component of the Notch signaling pathway, was associated with worse PFS and inferior distant metastasis free survival^20^. Hence, the association of *NOTCH3* mutations with worse PFS in our cohort further suggests its possible prognostic significance and warrants deeper investigation.

Multiple genetic mutations were associated with response to therapy in our recurrent HNSCC cohort. Mutations in *FGF4*, *SOX2*, *CCND1*, and *TP53* correlated with worse response following treatment with surgery, radiotherapy or chemotherapy. *SOX2* expression enhanced tumorigenicity in oral squamous cell carcinoma cell lines, and silencing *SOX2* increased the sensitivity of the cells to chemoradiation treatment^43^. Our findings similarly link *SOX2* mutations to worse response to chemotherapy, and *SOX2*-associated chemoresistance is reported across multiple malignancies including colorectal cancer, non-small cell lung carcinoma, melanoma, and ovarian cancers^44–47^. Notably, all recurrent treatments in *CCND1-*mutant patients resulted in progression, a finding consistent with overexpression of *CCND1* contributing to therapeutic resistance by promoting cell cycle progression and enhancing DNA damage repair, allowing tumor cells to recover from radiation-induced DNA damage and to bypass apoptotic checkpoints and continue proliferation despite exposure to cytotoxic chemotherapy, thereby reducing the efficacy of both radio- and chemo- therapies^48^. *CCND1* is reported as a predictive biomarker of treatment response in HNSCC; while low *CCND1* expression correlated with improved response to cisplatin-based neoadjuvant chemotherapy and better survival^49^, *CCND1* overexpression reduced sensitivity to chemotherapy and chemoradiotherapy, lower treatment response rates, and poorer clinical outcomes^50^, highlighting its potential role in therapeutic resistance.

In contrast, mutations in *MYL1*,* AXIN1*, and *ASXL1* were associated with improved treatment outcomes. Kang et al. 2024^51^ reported that mutations in epigenetic regulators such as ASXL1 can influence CD8⁺ T-cell states by promoting a stem-like memory phenotype characterized by increased self-renewal capacity and sustained antitumor activity during immunotherapy. However further studies are essential to determine how these mutations promote tumor sensitivity to treatments in recurrent HNSCC.

*TP53* mutations also emerged as a marker of poor response to both chemotherapy and immunotherapy. Osman et al. (2015)^53^ identified a subset of high-risk *TP53* mutations associated with decreased sensitivity to cisplatin. Prior studies have shown mixed associations between *TP53* mutations and immunotherapy outcomes, with some studies reporting that *TP53* mutations predict poor response to immunotherapy due to impaired immune response^54^, while certain *TP53* disruptive mutations predict improved response to immunotherapy^55^. Our findings align with the former, as *TP53-*mutated tumors demonstrated poor outcomes across treatment modalities. Although not significant, our findings from immunotherapy-specific analyses revealed that *TERT* mutations predicted poor response, Kouchkovsky et al.^56^ found *TERT* promoter mutations to be predictive of improved OS in patients treated with immunotherapy. Though the data is exploratory, the discrepancy in outcomes associated with mutations in *TERT* requires further analysis in a larger, independent recurrent HNSCC cohort.

Our decision-tree modeling added a further layer, illustrating how clinical and genomic factors may act jointly to shape therapeutic outcomes. For initial treatments, anatomic stage and tumor site were the strongest predictors of response. In the recurrent setting genomic drivers carried more weight, with mutations in *CCND1*, *TERT*, *CDKN2A*,* PIK3CA*, and *TP53* emerging as key determinants of treatment resistance or sensitivity. Gender also contributed to outcomes. Oral cavity tumors showed worse survival, particularly at advanced stages, compared to tumors at other sites, which is consistent with the existing literature^61^. We read these models as generating structured hypotheses about interacting predictors rather than as fixed decision rules.

Our study expands the current knowledge of genomic alterations in recurrent HNSCC; however, several limitations remain. Firstly, the analysis of recurrent events is limited by the inherent dependence of observations for the same patient. While we included multiple recurrent events for each patient in the treatment response analysis (Fig. 2) and PFS analysis (Fig. 3), we excluded any right-censored data points from PFS analysis to strengthen the associations that were identified. Additionally, the UMB recurrent cohort is relatively small, and the analysis of multiple recurrences from the same patient allows us to consider the entire spectrum of treatment, rather than the more-often studied initial treatment and response. Furthermore, although we identified mutations associated with prognosis and treatment response, functional assays were not performed to determine whether these mutations were pathogenic gain-of-function or loss-of-function events. Unraveling the downstream effects of these mutations could elucidate possible mechanisms of their effects on prognosis and therapeutic targets. Additionally, HPV status could not be analyzed due to the limited number of patients in our cohort, despite known biological differences between HPV + and HPV- disease. Furthermore, comparisons between our recurrent UMB cohort and the primary TCGA cohort should be interpreted with caution. These two cohorts differ in disease setting, since the UMB cohort comprises recurrent HNSCC while the TCGA cohort is predominantly treatment-naïve primary disease. They also differ in several clinically relevant characteristics that shape the genomic landscape of HNSCC, including HPV/p16 status, smoking history, race, and tumor subsite. Any of these factors may confound the apparent differences in mutational profile between the cohorts, such that alterations we attribute to the recurrent setting may in part reflect underlying differences in cohort composition. In addition, differences in sequencing platforms, panel design, and variant-calling pipelines between the two datasets may contribute further to technical variation that is difficult to separate from true biological differences. Another limitation includes our inability to perform fully adjusted analyses due to lack of complete and comparably coded data for these covariates across both cohorts and sample sizes within certain subgroups would not support stable multivariable modelling. The TCGA comparison should therefore be regarded as a descriptive benchmark that places our findings in the context of primary disease and consequently, these cross-cohort comparisons should be regarded as exploratory and hypothesis-generating. Further, the genomic features we report will need to be confirmed in studies in larger, independent, clinically matched recurrent HNSCC cohorts stratified by HPV status and other key clinical variables to define the molecular differences between recurrent and primary HNSCC.

## Conclusion

This study shows that recurrent HNSCC carries a distinct clinical and genomic landscape that appears to be associated with survival and treatment responsiveness. Mutations in *MTAP*, *LRP1B*, and *TP53* were associated with poorer survival outcomes, while mutations in *PIK3CA* correlated with more favorable outcomes in recurrent HNSCC. We also observed associations between treatment response and genetic alterations, with *FGF4*,* SOX2*,* CCND1*,* TERT* and *TP53* emerging as candidate contributors to therapeutic resistance. Decision tree modeling further suggested that the relative importance of specific genomic alterations, disease stage, and clinical factors may shift across initial and recurrent treatment attempts. Together, these findings support the integration of both genetic and clinical variables for optimizing and guiding individualized therapy and improving outcomes in recurrent HNSCC, though they remain hypothesis-generating. Larger, multi-institutional studies and functional validation of candidate mutations are warranted to further refine prognostic biomarkers and to determine whether any represent actionable therapeutic vulnerabilities.

## Supplementary Information

Below is the link to the electronic supplementary material.

Supplementary Material 1

Supplementary Material 2

## Data Availability

All data supporting the findings of this study are available in the paper and its Supplementary Information section. The results shown here are in part based upon data generated by the TCGA Research Network: https://www.cancer.gov/tcga. The datasets generated and analyzed during the current study are available in the dbGaP repository [https://www.ncbi.nlm.nih.gov/projects/gap/cgi-bin/study.cgi?study_id=phs004779, accession number: phs004779]. Treatment data are available in the supplementary excel file.

## Abbreviations

ASXL1: Additional sex combs-like transcriptional regulator 1
CBR: Clinical benefit rate
CCND1: Cyclin D1
CDKN2A: Cyclin dependent kinase inhibitor 2 A
CDKN2B: Cyclin dependent kinase inhibitor 2B
CHK1/2: Checkpoint kinase ½
CI: Confidence interval
CIC: Capicua transcriptional repressor
CR: Complete response
EGFR: Epidermal growth factor receptor
ENE: Extranodal extension
EP300: E1A-associated protein p300
FAT1: FAT atypical cadherin 1
FDA: Food and Drug Administration
FGF4: Fibroblast growth factor 4
FGFR3: Fibroblast growth factor receptor 3
HNSCC: Head and neck squamous cell carcinoma
HPV: Human papillomavirus
HR: Hazard ratio
KMT2D: Lysine methyltransferase 2D
LRP1B: Low density lipoprotein-related protein 1B
LVI: Lymphovascular invasion
MLH3: MutL Homolog 3
MTAP: Methylthioadenosine phosphorylase
MYC: Myelocytomatosis proto-oncogene
MYL1: Myosin light chain 1
NBN: Nibrin
NOTCH: Neurogenic locus notch homolog protein
NSD1: Nuclear receptor binding SET domain protein 1
OS: Overall survival
PD: Progression disease
PD-L1: Programmed death-ligand 1
PFS: Progression-free survival
PIK3CA: Phosphatidylinositol-4,5-bisphosphate 3-kinase catalytic subunit alpha
PLCG1: Phospholipase C Gamma 1
PLK1: Polo-like kinase 1
PNI: Peri-neural invasion
PR: Partial response
R/M: Recurrent or metastatic
SD: Stable disease
SOX2: Sex determining region Y-Box 2
SRC: Sarcoma proto-oncogene
TCGA: The Cancer Genome Atlas
TERT: Telomerase reverse transcriptase
TGF-beta: Transforming growth factor-beta
TP53: Tumor protein 53
UMB: University of Maryland Baltimore
WES: Whole exome sequencing
ZNF750: Zinc finger 750
