# Supplementary material for: Tumor genomic alterations and their exploratory association with treatment response in recurrent head and neck cancer patients
Source: Sci Rep. 2026 Sep 9;16:36879. doi: 10.1038/s41598-026-68381-1 (PMC13736499; doi:10.1038/s41598-026-68381-1)
Supplement: Supplementary file 1 [file 41598_2026_68381_MOESM1_ESM.docx]

**Supplementary Figures**

 **Supplementary Figure 1. Clinical history for the UMB cohort.** Swimmer plot showing treatment type, response to treatment, and five-year overall survival of individual patients with respect to time since diagnosis between patients that passed away (red) and patients alive at five years or no overall survival information available (blue). Patients are ordered by time until censorship.

**Supplementary Figure 2. Progression Free Survival for Chemotherapy and Initial Treatment of UMB Cohort.** Kruskal-Wallis H test indicates differences in progression-free time in days on the Y-axis in relation to **(A)** treatment type for the initial treatment only (Kruskal-Wallis H-test p-value = 5.4299E-1), **(B)** response to the initial treatment, with significant pairwise p-values shown on the plot (Kruskal-Wallis H-test p-value = 1.7098E-9), **(C)** chemotherapy given as initial treatment, compared to chemotherapy given as treatment for recurrent disease (Kruskal-Wallis H-test p-value = 6.6354E-4).

**Supplementary Figure 3.** Bar plot indicating which types of treatment were given for each line of therapy.

**Supplementary Figure 4. Univariate Cox regression analysis of gene mutations for survival outcomes in recurrent HNSCC.** **(A).** Univariate Cox regression analysis of gene mutations for overall survival. **(B).** Univariate Cox regression analysis for disease-free survival.

**Supplementary Tables**

**Supplementary Table 1. Significant Mutated Genes in Response to Treatment**

| **Gene** | **All Treatment Types** | **Surgery and/or Radiation** | **Chemotherapy** | **Immunotherapy** | **Better or worse for response** |
| --- | --- | --- | --- | --- | --- |
| TP53 | p=0.0067 | p=0.0703 | p=0.003 |  | Worse |
| SOX2 | p=0.0376 | p=0.0304 | p=0.0433 |  | Worse |
| CCND1 | p=0.0588 | p=0.0197 |  |  | Worse |
| FGF4 | p=0.0807 | p=0.0124 |  |  | Worse |
| ASXL1 | p=0.0101 |  |  | p=0.0289 | Better |
| AXIN1 | p=0.0209 |  |  | p=0.0831 | Better |
| MLH3 | p=0.0405 |  |  |  | Better |
| NSD1 | p=0.0405 |  |  |  | Better |
| MYL1 | p=0.0492 |  |  |  | Better |
| FGFR3 | p=0.0597 |  |  |  | Better |
| HNF1A | p=0.0765 |  |  |  | Better |
| NBN | p=0.0765 |  |  |  | Better |
| CYLD | p=0.082 |  |  | p=0.0679 | Better |
| BRCA1 | p=0.0872 |  |  |  | Better |
| SPOP | p=0.0872 |  |  |  | Better |
| EGFR |  | p=0.059 |  |  | Worse |
| FGF3 |  | p=0.0626 |  |  | Worse |
| TERT |  |  |  | p=0.0854 | Worse |
| AXIN2 |  |  |  | p=0.0831 | Better |
| LRP1B |  |  |  | p=0.0831 | Better |
